# Nutritional Factors and Arrhythmic Risk in Long QT Syndrome: A Narrative Review of Mechanistic and Clinical Evidence

**DOI:** 10.1016/j.advnut.2025.100525

**Published:** 2025-09-22

**Authors:** Andrea Mazzanti, Matteo Floriano, Deni Kukavica, Alessandro Trancuccio, Silvia G Priori

**Affiliations:** 1 Unit of Molecular Cardiology, Istituti Clinici Scientifici Maugeri, IRCCS, Pavia, Italy; 2 Department of Molecular Medicine, University of Pavia, Pavia, Italy

**Keywords:** long QT syndrome, dietary supplements, grapefruit juice, energy drinks, hERG channel, potassium, Andersen-Tawil syndrome, Timothy syndrome, QT interval, hypoglycemia

## Abstract

Long QT syndrome (LQTS) is an umbrella term for a group of genetic cardiac channelopathies characterized by prolonged ventricular repolarization and increased risk of life-threatening arrhythmias. Although β-blockers and lifestyle modifications remain central to management, specific dietary components may influence repolarization and arrhythmic risk, particularly in genetically predisposed individuals. This review summarizes mechanistic and clinical evidence on the electrophysiological effects of selected nutrients, food constituents, and supplements—including grapefruit juice, licorice, over-the-counter products, and energy drinks. Gene–nutrient interactions and their impact on ion channel function, drug metabolism, and electrolyte balance are discussed. The second part of the review outlines genotype-specific considerations, such as potassium supplementation and dietary guidance for rare forms of LQTS, including Andersen-Tawil and Timothy syndromes. Clinical data are presented in tabular format to facilitate interpretation. By integrating mechanistic and clinical data, the review aims to support dietary counseling and inform clinical decision making in the management of LQTS.

**Statement of Significance**

This is the first review, to our knowledge, to analyze and summarize current mechanistic, clinical, and case-based evidence on the impact of dietary exposures on arrhythmic risk in long QT syndrome. Through interdisciplinary engagement, this review aims to provide a structured overview of the interactions between nutritional factors and arrhythmic risk in long QT syndrome, with practical implications for care. Specifically, we review evidence that may be used for genotype-specific guidance and which may serve to propose nutrition as an underutilized axis of arrhythmia prevention.

## Introduction

Long QT syndrome (LQTS) encompasses a group of inherited cardiac channelopathies characterized by prolonged ventricular repolarization, which predisposes affected individuals to life-threatening arrhythmias such as torsade de pointes (TdP) and ventricular fibrillation (VF), potentially resulting in sudden cardiac death (SCD) [1]. Clinically, repolarization duration is assessed using the corrected QT interval (QTc), which adjusts for heart rate and serves as a key marker for diagnosis and assessment of arrhythmic risk in LQTS. Standard management includes β-blockers (βB) and lifestyle changes [1].

Recent attention has focused on how nutrition affects ion channel function, electrolyte homeostasis, and drug metabolism. However, the nutritional aspects of lifestyle management in patients with LQTS remain particularly poorly defined, and advising patients on lifestyle modifications is challenging as the data are often scarce and most indications are based on individual experience. This assumes particular relevance as lifestyle modifications play a fundamental role in the management of LQTS as patients should limit the exposure to arrhythmic triggers. This review summarizes mechanistic and clinical evidence linking nutrition and QT modulation to support dietary recommendations for individuals with LQTS. It is divided into 2 parts: *1*) proarrhythmic foods, supplements, and beverages and *2*) nutritional influences across LQTS genotypes with implications for personalized management.

### A concise overview of LQTS

Congenital LQTS, estimated to affect 1 in 2500 individuals, is caused by mutations on genes encoding for cardiac ion channels [1]. In 70% of LQTS cases, loss-of-function mutations affect genes encoding for cardiac potassium channels, particularly K_V_7.1 [type 1 LQTS (LQT1), *KCNQ1* gene [2]], K_V_11.1 or human ether-à-go-go-related gene (hERG) (LQT2, *KCNH2* gene [3]), and K_ir_2.1 (LQT7, *KCNJ2* gene [4]). These mutations reduce the repolarizing potassium currents—I_Ks_, I_Kr_, and I_K1_—and prolong ventricular repolarization, thus reducing the so-called “repolarization reserve.” This term refers to the intrinsic safety margin that enables the myocardium to buffer physiologic or pharmacologic perturbations without triggering arrhythmias. When this reserve is compromised, as in LQTS, even modest changes in electrolytes, ion channel function, or drug exposure may provoke life-threatening arrhythmias. Rare variants of LQTS (5%–10% of cases) arise from gain-of-function mutations in the genes encoding the Na_V_1.5 sodium channel (LQT3, *SCN5A* gene [5]) and the Ca_V_1.2 Ca^2+^ channel (LQT8, *CACNA1C* gene [6]). These mutations impair the inactivation of the sodium current (I_Na_) and calcium current (I_Ca_), respectively, resulting in a sustained depolarizing inward current that prolongs the action potential duration at the cellular level and extends the QT interval at the organ level.

βB therapy is the mainstay of pharmacological therapy for LQTS and is indicated in all patients with LQTS, including those with a genetic diagnosis and normal QTc [1]. In patients with potassium channel mutations (particularly, LQT1 and LQT2), in whom fatal arrhythmias are triggered by exercise or emotional stress, βBs are used to counteract the effect of catecholamines [7,8]. In these LQTS subtypes, β-adrenergic stimulation is deleterious as potassium currents are pivotal for exercise-induced QT interval shortening, and nonselective β-adrenergic blockade has shown excellent results [9]. In contrast, arrhythmias in LQT8 and LQT3 have fewer links to adrenergic activation, with a more modest response to βB therapy [10]. Given that the duration of the QTc interval is one of the most potent predictors of arrhythmic events [9,11], our group has pioneered a substrate-oriented approach in patients with LQT3 [12,13]. We showed that mexiletine, a sodium channel blocker that contrasts the effects of gain-of-function mutations causing LQT3, abbreviated and normalized the pathologically prolonged QTc interval, translating into a clinically meaningful benefit by reducing the number of patients with arrhythmic events, the number of events per patient, and the annual event rate [13]. Finally, the use of an implantable cardioverter defibrillator (ICD) is indicated in all survivors of a cardiac arrest and patients who continue experiencing arrhythmic symptoms (i.e., syncope or hemodynamically nontolerated ventricular arrhythmias) while receiving adequate medical therapy [1].

## Dietary Factors That Can Prolong the QT Interval

Although delayed ventricular repolarization in LQTS primarily results from pathogenic variants affecting cardiac ion channels, its severity—and arrhythmic risk—can be modulated by external elements, including medications, electrolyte disturbances, and autonomic fluctuations. Among these, nutritional factors interfere with cardiac repolarization through several mechanisms, including ion channel inhibition, disturbances of electrolyte balance, modulation of autonomic tone, and alteration of drug metabolism.

Such effects are amplified in patients with reduced repolarization reserve—those whose myocardium cannot buffer additional stressors [14]. In this setting, even mild dietary influences may provoke clinically meaningful QT prolongation and promote TdP [15].

The sections below examine key dietary contributors to QT prolongation, mechanistic underpinnings, and relevance for managing LQTS.

### Grapefruit juice

Grapefruit (*Citrus paradisi*) juice has emerged as a clinically relevant dietary contributor to QT prolongation, due to its content of flavonoids—particularly naringenin—and furanocoumarins such as bergamottin [16]. These compounds interfere with cardiac repolarization through 2 mechanisms: direct ion channel inhibition and alteration of drug metabolism [15].

#### Molecular mechanisms

*In vitro* studies have demonstrated that naringenin inhibits the hERG-mediated I_Kr_ current, with IC_50_ values of 36.5 μM in HEK293 cells and 102.3 μM in *Xenopus* oocytes, leading to prolonged ventricular repolarization [17]. Furthermore, naringenin also inhibits K_ir_2.1, Na_V_1.5, and Ca_V_1.2 channels with IC_50_ values ranging from 30 to 100 μM, potentially compounding arrhythmic risk [18]. Other flavonoids present in grapefruit juice, such as hesperetin, morin, flavones, and kaempferol, have also been shown to modulate hERG activity, further supporting the potential arrhythmogenic effects of grapefruit juice and its components [17]. Importantly, multiple flavonoids in grapefruit juice exert additive or synergistic effects on hERG blockade [17].

Beyond ion channel inhibition, grapefruit juice is a potent inhibitor of cytochrome P450 3A4 (CYP3A4), primarily because of its furanocoumarin content. CYP3A4 is responsible for the metabolism of numerous QT-prolonging drugs, including amiodarone, sotalol, and macrolide antibiotics [19,20]. By inhibiting CYP3A4, grapefruit juice can increase plasma concentrations of these medications, effectively enhancing their QT-prolonging effects [21].

Of note, the flavonoid composition of grapefruit juice is enhanced when ripening fruit is exposed to freezing temperatures and is at a peak during early development. In producing grapefruit juice, forceful mechanical compression increases the presence of compounds derived from fruit tissues high in naringenin, as contrasted with grapefruit juice obtained solely from juice vesicles by hand-squeezed fruit without pulp [22].

#### Clinical evidence

Table 1 summarizes clinical evidence for the modulatory effects of grapefruit juice. Benton et al. [21] demonstrated that 240 mL of grapefruit juice consumed with terfenadine, a QT-prolonging drug, led to a significant increase in terfenadine plasma levels and an increase in QT interval from 420 ms to 434 ms (*P* < 0.05). This finding highlighted the role of CYP3A4 inhibition by grapefruit juice, which enhances the bioavailability of QT-prolonging drugs.

**TABLE 1:** Clinical evidence of QT prolongation induced by juice from grapefruit and other citrus fruits.

Reference	Type of article	Sex	Age (y)	Type of LQTS	Nutritional exposure	QT/arrhythmia outcome
Hermans et al. (2003) [23]	Case report (LoE C)	Female	33	Noncongenital LQTS	Large amounts of tonic water and grapefruit juice	TdP and QTc up to 580 ms; QTc normalized to 450 ms after 2 d
Zitron et al. (2005) [17]	Clinical trial (LoE C)	5 male, 5 female	25.2 ± 4.6	Noncongenital LQTS	1 L of grapefruit juice	+10.6 ± 2.8 ms (t = 4 h); +12.5 ± 4.2 ms (t = 5 h); normalization by 9 h
Piccirillo et al. (2008) [25]	Controlled clinical trial (LoE C)	7/3 (DCM); 8/4 (HCM); 7/3 (healthy)	DCM: 60 ±5; HCM: 62 ± 3; Healthy: 61 ± 4	Noncongenital LQTS	1 L of grapefruit juice	QTc Placebo (ms): DCM: 432 ± 38, HCM: 422 ± 31, Healthy: 348 ± 12; QTc Grapefruit: DCM: 446 ± 40, HCM: 438 ± 37, Healthy: 362 ± 16
Chorin et al. (2019) [26]	Randomized clinical trial (LoE B)	Controls: 17 female/male; LQTS: 5 female/5 male	Controls: 29.8 ± 8.7; LQTS: 45.7 ± 13.6	LQT1, *n* = 3; LQT2, *n* = 5; LQT3, *n* = 2	Controls: 2 L of grapefruit juice patients with LQTS: 1.5 L	Controls: +14.0 ms (95% CI: 6, 22 ms); LQTS: +21.8 ms (95% CI: 3, 35 ms)
Persia-Paulino et al. (2020) [24]	Case report (LoE C)	Male	52	LQT1	Chronic intake of 1–2 L/d of orange and lemon juice for ∼1 y	QTc increased from 470–500 ms to 638 ms; notched T waves appeared.

Abbreviations: CI, confidence interval; DCM, dilated cardiomyopathy; HCM, hypertensive cardiomyopathy; LoE, level of evidence; LQT1/2/3, type 1/2/3 long QT syndrome; LQTS, long QT syndrome; QTc, corrected QT interval; TdP, torsade de pointes.

In 2003, Hermans et al. [23] described a diabetic female with congenital LQTS who experienced severe QTc prolongation (up to 590 ms) and TdP after consuming “large amounts” of grapefruit juice and tonic water containing quinine. Her QTc interval normalized to 450 ms upon cessation of intake of these beverages, illustrating the additive arrhythmic potential of grapefruit juice when combined with other QT-prolonging agents like quinine. A similar observation was made by Persia-Paulino et al. [24], who reported a patient with LQT1 who experienced marked QTc prolongation (from 471 ms to 638 ms) and dizziness after chronic intake (1–2 L/d) of lemon and orange juices. QTc reduced over 6 d after cessation, suggesting that citrus-derived flavonoids beyond grapefruit may pose a risk in predisposed individuals.

Subsequent data from a clinical trial conducted by Zitron et al. [17] showed 10 healthy volunteers who consumed 1 L of grapefruit juice (containing >1000 μmol/L naringenin) had a significant QTc prolongation of 12.5 ± 4.2 ms (*P* = 0.02), demonstrating grapefruit juice’s ability to modulate cardiac repolarization in vivo. Importantly, this effect opposed the typical diurnal trend of QTc shortening in the late morning, suggesting a true pharmacologic action rather than physiological variability.

Further supporting these findings, Piccirillo et al. [25] investigated the effects of the consumption of 1 L of pink grapefruit juice in 32 participants (10 healthy volunteers and 22 patients with dilated or hypertensive cardiomyopathy). QTc prolongation was observed in all groups, with increases of +14 ms in dilated cardiomyopathy (to 446 ± 40 ms), +16 ms in hypertensive patients (to 438 ± 37 ms), and +14 ms in healthy individuals (to 362 ± 16 ms), comparable to the effects of sotalol and amiodarone, 2 well-known QT-prolonging drugs.

Finally, Chorin et al. [26] evaluated the QT-prolonging effects of grapefruit juice in 30 healthy volunteers and 10 patients with LQTS. Healthy participants consuming 2 L of grapefruit juice experienced a mean QTc prolongation of 14.0 ms [95% confidence interval (CI): 6.2, 21.7 ms; *P* < 0.001], comparable to the effects of moxifloxacin, a QT-prolonging drug. Of interest, patients with LQTS exhibited a greater QTc increase of 21.8 ms (95% CI: 3.4, 35.3 ms; *P* = 0.034), with significant QTc prolongation observed even after consuming 1 L of grapefruit juice.

**Conclusions**

Grapefruit juice contributes to significant QT prolongation via 2 distinct mechanisms: flavonoid-mediated direct hERG inhibition and furanocoumarin-induced CYP3A4 inhibition, which enhance the systemic exposure of QT-prolonging drugs.

Although the resulting QTc prolongation is generally modest in healthy individuals, these mechanisms can synergize in vulnerable populations, significantly increasing arrhythmic risk.

**Clinical implications in patients with LQTS**

Grapefruit juice intake should be assessed in patients presenting with unexplained QT prolongation or arrhythmias, and evidence suggests that high intake (>1 L/d) should be avoided.

### Licorice

Licorice (*Glycyrrhiza glabra*) is a widely used ingredient in candies, herbal medicines, and teas. Its bioactive compounds have been associated with hypertension, hypokalemia [27,28] and QT interval prolongation through both electrolyte disturbances [29] and direct effects on cardiac ion channels [30].

#### Molecular mechanisms

Glycyrrhizin, a compound found in licorice, and more specifically its bioactive metabolite glycyrrhetinic acid, inhibits 11β-hydroxysteroid dehydrogenase type 2. This inhibition results in impaired inactivation of cortisol, leading to inappropriate stimulation of mineralocorticoid receptors and subsequent aldosterone-like effects, defined as pseudohyperaldosteronism: sodium retention and hypertension, and potassium excretion with hypokalemia [28,31]. On the contrary, liquiritigenin, a flavonoid contained in licorice, has been shown to directly inhibit the hERG potassium channel, independent of electrolyte disturbances; in vitro studies have shown moderate hERG inhibition, with an EC_50_ of 53.3 μM [30,32].

#### Clinical evidence

A review of 12 reported cases of licorice-induced QT prolongation and arrhythmias (Table 2) reveals that most of the affected individuals were female (75%, 9/12), with a mean age of 63 y (range: 34–95 y). Hypokalemia, defined as potassium levels <3.5 mEq/L, was present in 92% (11/12) of cases, with values ranging from 1.4 to 2.9 mEq/L. Furthermore, most patients had underlying comorbidities, including hypertension and coronary artery disease. This suggests that predisposing cardiovascular conditions may amplify risk of arrhythmias in patients exposed to licorice. High levels of licorice intake have often been implicated in these cases. Eriksson et al. [33] described a 44-y-old female who consumed 40–70 g/d of licorice candy for 4 mo, leading to hypokalemia (2.3 mEq/L), QT interval prolongation (QTc 510 ms), and an episode of TdP. Yoshida et al. [34] reported a case of an 84-y-old female with Takotsubo cardiomyopathy who consumed a multi-herb extract containing licorice for over a decade and developed polymorphic ventricular tachycardia, with a fatal outcome. In addition to chronic intake, noxious effects of acute excessive consumption have also been documented. Specifically, Zhang et al. [35] described a 34-y-old female who ingested 400 licorice tablets in 1 wk, presenting with TdP, hypokalemia (1.9 mEq/L), and a QTc of 540 ms. Although most cases involve hypokalemia, licorice-induced QT prolongation can occur even in its absence. Oztürk et al. [36] described a 59-y-old female who developed TdP with a QTc of 600 ms after consuming 5–6 glasses of licorice tea daily for 2 d, despite having normal potassium levels. Patel et al. [37] similarly documented QT prolongation in a patient taking deglycyrrhizinated licorice supplements, suggesting that other bioactive compounds in licorice may also contribute to its arrhythmogenic effects. The risk is further amplified by concurrent QT-prolonging drugs, as shown by Miyamoto et al. [38], who reported TdP in an 82-y-old male taking garenoxacin and disopyramide alongside licorice-containing herbal medicines.

**Conclusions**

Licorice is an underrecognized contributor to QT prolongation and arrhythmias. Its proarrhythmic effects result from both glycyrrhizin-induced hypokalemia and flavonoid-mediated hERG inhibition. Although modest doses of licorice are not expected to cause clinically relevant QT interval prolongation in healthy individuals, significant prolongation may occur with higher intake or in those with LQTS.

**Clinical implications in patients with LQTS**

Assess licorice intake in individuals with unexplained QT prolongation, hypokalemia, or arrhythmic events. Evidence suggests that excessive licorice consumption should be avoided, particularly when combined with QT-prolonging drugs.

**TABLE 2:** Case reports of licorice-induced QT prolongation and arrhythmic outcomes.

Reference	Patient details	Nutritional exposure	QT/arrhythmia outcome
Eriksson et al. (1999) [33] (LoE C)	Female, 44 y, no major comorbidities	40–70 g/d of candy for 4 mo	QTc 450–510 ms, TdP, hypokalemia (2.3 mEq/L) Recurrent ventricular extrasystoles
Crean et al. (2009) [39] (LoE C)	Female, 71 y, hypertension, coronary artery disease, mild kidney failure (eGFR 55 mL/min/1.73 m^2^)	Large amounts of candy (quantity unspecified)	QTc 566 ms, cardiac arrest, VF, hypokalemia (1.6 mEq/L)
Miyamoto et al. (2010) [38] (LoE C)	Male, 82 y, pneumonia, prostate cancer, depression	Herbal medicines with ∼0.78 g/d of licorice	Hypokalemia (2.3 mEq/L); QTc 640 ms, VT, TdP after drug initiation
Oztürk et al. (2013) [36] (LoE C)	Female, 59 y, coronary artery disease, mild septal hypertrophy	5–6 glasses/d of licorice tea for 2 d	QTc 600 ms, TdP, potassium normal (3.7–4.2 mEq/L)
Panduranga et al. (2013) [40] (LoE C)	Female, 38 y, obesity, hypertension, postpartum 6 mo	Licorice tea, 3 times/d for 2 mo	QTc 485 ms, hypokalemia (2.4 mEq/L), TdP requiring 40 ICD shocks
Smedegaard et al. (2019) [41] (LoE C)	Female, 43 y, hypertension and myxedema	70 g/d licorice and unknown amount of licorice tea	QTc 476 ms, hypokalemia (1.9 mEq/L)
Patel et al. (2021) [37] (LoE C)	Male, 61 y, hypertension and fibromyalgia	20 deglycyrrhizinated licorice tablets/d	QTc 670 ms, flattened T waves, hypokalemia (1.9 mEq/L)
Yoshida et al. (2022) [34] (LoE C)	Female, 84 y, Takotsubo cardiomyopathy, previous stroke, Alzheimer-type dementia	Multi-herb extract containing licorice for 10+ y	QTc 464 ms, TdP, hypokalemia (1.4 mEq/L), fatal outcome due to Takotsubo cardiomyopathy
Molina-Lopez et al. (2023) [42] (LoE C)	Male, 95 y, T2DM, stage 4 CKD, anemia of chronic disease, hypothyroidism	Daily licorice candy twists and tea for >2 mo	QTc 534 ms, TdP, hypokalemia (2.3 mEq/L)
Han et al. (2023) [29] (LoE C)	Female, 89 y, no major comorbidities	Herbal medicine with licorice for 2 mo	QTc 686 ms, hypokalemia (1.5 mEq/L), polymorphic ventricular tachycardia requiring >30 ICD shocks
Kawatani et al. (2023) [43] (LoE C)	Female, 50 y, hypertension, hysterectomy, depression	7.5 g/d of herbal medicine with licorice for 1 y Conversion amounts: Licorice: 6 g/d Glycyrrhizin: 240 g/d	QTc 630 ms, TdP, hypokalemia (3.4 → 2.9 mEq/L)
Zhang et al. (2024) [35] (LoE C)	Female, 34 y, depression and mania	400 licorice tablets over 1 wk	QTc 540 ms, TdP, hypokalemia (1.9 mEq/L)

Abbreviations: CKD, chronic kidney disease; eGFR, estimated glomerular filtration rate; ICD, implantable cardioverter-defibrillator; LoE, level of evidence; QTc, corrected QT interval; T2DM, type 2 diabetes mellitus; TdP, torsade de pointes; VF, ventricular fibrillation; VT, ventricular tachycardia.

### Quinine

Quinine, a bitter alkaloid obtained from the bark of the *Cinchona* tree, is often used as a flavoring agent in tonic water and has pharmacological applications, particularly as an antimalarial drug [44,45]. Although it is generally safe at low concentrations, quinine has been associated with cardiac conduction abnormalities and QT interval prolongation, particularly at supratherapeutic doses or in predisposed individuals [45,46].

#### Molecular mechanisms

Quinine exerts its electrophysiological effects through multichannel inhibition, principally targeting I_Kr_ and the fast-inward I_Na_, and, to a lesser extent, the transient outward potassium current (I_to_).

In heterologous expression systems, quinine blocks hERG-encoded I_Kr_ in a concentration- and voltage-dependent manner, with an estimated IC_50_ of 44–98 μM, markedly less potent than its stereoisomer quinidine [[47], [48], [49]]. This inhibition can prolong the ventricular action potential under appropriate conditions, particularly when quinine accumulates, or the repolarization reserve is compromised. Quinine also inhibits peak I_Na_, contributing to slowed conduction and increased ventricular refractoriness [50]. Because I_Na_ inhibition primarily affects depolarization and conduction velocity, its direct contribution to QT prolongation is likely modest, but it may enhance arrhythmia susceptibility by promoting dispersion of repolarization and facilitating reentrant circuits, especially in the setting of pre-existing conduction slowing [44]. Clinical evidence of I_Na_ inhibition is supported by quinine-induced QRS widening on surface electrocardiogram (ECG) and prolongation of conduction intervals, consistent with its classification as a class I antiarrhythmic agent [50].

Finally, quinine inhibits I_to_ with IC_50_ values in the range of 11–15 μM, as demonstrated in patch-clamp studies in isolated rat ventricular myocytes [51]. Although the role of I_to_ in human ventricular repolarization is limited compared with I_Kr_, its inhibition may contribute to early repolarization prolongation in specific myocardial regions, such as the right ventricular epicardium.

Overall, quinine’s proarrhythmic potential is mechanistically explained by a combination of hERG-mediated I_Kr_ inhibition, which delays phase 3 repolarization, and I_Na_ blockade, which prolongs depolarization and may indirectly affect repolarization dynamics. I_to_ inhibition likely plays a minor but contributory role in certain myocardial regions. At therapeutic concentrations, the net effect is usually modest, but it may become clinically significant in the presence of additional QT-prolonging agents, electrolyte imbalances, or congenital channelopathies.

#### Clinical evidence

Although quinine’s effects on repolarization are modest under most conditions, multiple case reports have linked it to QTc prolongation and TdP, particularly in the context of polypharmacy or excessive intake.

Sheehan et al. [44] reported a 91-y-old female who experienced extreme QTc prolongation (up to 800 ms) and TdP following exposure to multiple quinine sources, including hydroxychloroquine for rheumatoid arthritis, quinine sulfate for leg cramps, and nightly gin and tonic. Discontinuation of quinine sources resulted in normalization of the QT interval and complete resolution of symptoms.

In 2003, Hermans et al. [23] described a 33-y-old female with LQTS who experienced significant QTc prolongation (up to 580 ms) and TdP after consuming large amounts of tonic water containing quinine combined with grapefruit juice. Her symptoms resolved after discontinuing both substances.

These cases illustrate the dose-dependent risks of quinine, particularly when combined with other QT-prolonging agents. Excessive consumption of quinine-containing beverages (e.g., >1 L/d) [45] or simultaneous exposure to quinine-containing drugs [44] substantially increases the likelihood of arrhythmic events.

Regulatory authorities, including the European Food Safety Authority, have issued warnings advising that individuals with cardiac arrhythmias or predisposing conditions should only consume quinine-containing products under medical supervision [45]. The United Kingdom Medicines and Healthcare products Regulatory Agency similarly recommends caution because of quinine’s dose-dependent QT-prolonging effects [52].

**Conclusions**

Quinine is a recognized risk factor for QT prolongation, via multichannel inhibition, particularly at high doses or when combined with QT-prolonging agents. Although low doses are generally safe for healthy individuals, excessive intake may increase the risk of severe events such as TdP.

**Clinical implications in patients with LQTS**

Investigate quinine exposure in patients with QT prolongation or unexplained arrhythmias. Evidence suggests that intake of quinine-containing products, including tonic water, should be limited whenever possible.

### Food supplements with QT-prolonging effects

Certain dietary supplements pose significant risks for QT interval prolongation (Table 3). Despite their perceived safety and widespread use, these supplements often contain bioactive compounds that interfere with cardiac ion channels or drug metabolism. Unlike pharmaceuticals, dietary supplements are usually not subject to rigorous safety evaluations, leaving critical gaps in understanding their arrhythmic potential.

**TABLE 3:** QT-prolonging effects of dietary supplements: selected case reports.

Reference	Sex	Age (y)	Type of LQTS	Nutritional exposure	QT/arrhythmia outcome
Saliba et al. (2001) [69] (LoE C)	Female	47	Not congenital LQTS	Cesium chloride, green tea with licorice, citrus flavonoids supplement	QTc = 691 ms, polymorphic VT, hypokalemia (3.2 mEq/L)
Dalal et al. (2004 [70] (LoE C)	Female	43	Not congenital LQTS	Cesium chloride (9 g/d for 10 d)	QTc = 624 ms, VT, hypokalemia (3.1 mEq/L)
Vyas et al. (2006) [65] (LoE C)	Female	39	Not congenital LQTS	Cesium chloride, other unspecified supplements; 3.7–7.5 L of water/d	QTc = 616 ms, syncope, hypokalemia (3.1 mEq/L), hypomagnesemia (1.4 mg/dL)
Horn et al. (2015) [68] (LoE C)	Male	45	Not congenital LQTS	Cesium chloride (3 g/d, oral and topical)	Hypokalemia (nadir 3.3 mEq/L), no QTc reported, no arrhythmia documented
Kashiwa et al. (2021 [57] (LoE C)	Female	24	Type 1 LQTS (*KCNQ1* T587M)	Estrogenic supplement (*Pueraria mirifica*) for 1 wk	QTc = 674–764 ms, VF, refractory TdP. QTc, normalized to 439 ms after 8 d
Déléaval et al. (2022) [59] (LoE C)	Female	56	Not congenital LQTS	Hemp oil (CBD/CBG); berberine 250 mg/d	QTc = 667 ms, TdP, normalization to 413 ms after 5 d

Abbreviations: CBD, cannabidiol; CBG, cannabigerol; LoE, level of evidence; LQTS, long QT syndrome; QTc, corrected QT interval; TdP, torsade de pointes; VF, ventricular fibrillation; VT, ventricular tachycardia.

In 2016, Nguyen et al. [53] reviewed 26 studies evaluating the effects of dietary supplements on QT intervals. Of these studies, >30% reported electrocardiographic changes, underscoring the potential risks. In several cases, however, limitations such as small sample sizes, lack of control groups, and inconsistent ECG monitoring emphasize the need for standardized safety protocols [53].

#### Pueraria mirifica

*Pueraria mirifica*, a Southeast Asian herbal supplement marketed for menopausal symptom relief, contains phytoestrogens such as miroestrol and deoxymiroestrol. Initially widely used in native Southeast Asia, in recent years, it has spread worldwide as it is marketed as a natural alternative to estrogen therapy for menopause. However, despite its wide availability, also online, neither the European Medicine Agency nor the United States Food and Drug Administration (FDA) regulates supplements, limiting the ability to estimate dose-response relationships or risk thresholds. This assumes relevance because, although direct electrophysiological studies on their cardiac effects are lacking, their structural and functional similarity to estradiol suggests potential interaction with cardiac ion channels.

Estradiol is known to prolong the QT interval by activating L-type Ca^2+^ channels and inhibiting multiple cardiac K_V_ currents [54], including the rapid delayed rectifier potassium current I_Kr_, through suppression of hERG channel activity in a receptor-independent manner [55]. In addition, estrogen may impair the slow delayed rectifier current I_Ks_ by downregulating KCNE1 mRNA, further reducing repolarization reserve [56]. KCNE1 encodes the β-subunit of the I_Ks_ channel complex, which assembles with K_V_7.1 to control its gating and amplitude.

The relevance of these mechanisms is exemplified by a recent case describing the first gene-supplement interaction involving *Pueraria mirifica* [57]. A 24-y-old female carrying the *KCNQ1* T587M mutation—associated with impaired trafficking of both K_V_7.1 (I_Ks_) and hERG channels [58]—developed marked QT prolongation, VF, and refractory TdP after 1 wk of supplement intake. In this setting, estrogenic stimulation by phytoestrogens likely acted synergistically with the genetic defect by further suppressing I_Kr_, thereby critically compromising repolarization reserve. The patient presented with a QTc of up to 764 ms and experienced multiple episodes of TdP requiring electrical shocks. Upon discontinuation of the supplement and supportive therapy, QTc gradually normalized to 439 ms within 8 d [64].

#### Berberine

Berberine, a bioactive alkaloid extracted from Coptis root and *Phellodendron* Chinese, has been widely used in traditional Chinese and Ayurvedic medicine for type 2 diabetes mellitus, hyperlipidemia, and hypertension. Akin to *Pueraria mirifica*, berberine has recently gained popularity as an over-the-counter supplement, widely accessible through commercial and online platforms. Despite its popularity, berberine has been shown to inhibit hERG potassium channels [[59], [60], [61]], which is rarely disclosed in product literature.

This proarrhythmic potential was highlighted in a case report by Déléaval et al. [59], describing a 56-y-old female who developed a markedly prolonged QTc (667 ms) and TdP after consuming 250 mg berberine /d alongside high doses of hemp oil containing cannabinoids. Her QTc normalized within 5 d after discontinuing all supplements, underscoring the reversible yet significant arrhythmic risk associated with berberine use.

#### Cannabinoids

Cannabinoids, particularly cannabidiol (CBD) and cannabigerol, have gained popularity for their claimed therapeutic effects; however, both exhibit electrophysiological actions relevant to arrhythmogenesis. Experimental studies in guinea pig models showed that CBD inhibited hERG channels and prolonged the action potential at concentrations of 1–5 μM [62]. CBD also inhibits CYP isozymes, including CYP3A4 and CYP1A2, which can lead to an elevated systemic concentration of coadministered QT-prolonging compounds [59,63].

#### Cesium chloride

Cesium chloride, marketed as an alternative cancer therapy, has been flagged by the FDA for severe cardiac risks, including hypokalemia and arrhythmia [64]. Mechanistically, cesium blocks both inward rectifying potassium channels (K_ir_) and hERG channels, leading to delayed repolarization and depolarization abnormalities [65,66]. In addition to direct potassium channel blockade, cesium intake has been associated with the depletion of both intracellular and extracellular potassium [67], with urinary potassium wasting being proposed as the mechanism of cesium-associated hypokalemia [68].

Several case reports have documented the arrhythmogenic effects of cesium chloride, including QT prolongation and polymorphic ventricular tachycardia. First, Saliba et al. [69] described a 47-y-old female with QTc prolongation to 691 ms and polymorphic ventricular tachycardia after taking cesium chloride along with licorice tea and citrus flavonoid supplement, with resolution upon discontinuation. Additional cases by Dalal et al. [70] and Vyas et al. [65] reported QTc prolongation and hypokalemia after cesium chloride use, also resolving after cessation.

More recently, Horn et al. [68] described a 45-y-old male with metastatic cancer who developed hypokalemia attributed to urinary potassium wasting while self-administering cesium chloride. Although no arrhythmias or QT prolongation were documented in this patient, the case provided important mechanistic insight, demonstrating that amiloride therapy can mitigate cesium-induced kaliuresis [73]. The same report includes a comprehensive summary of prior cases in which cesium use was associated with QT prolongation, TdP, or ventricular tachycardia.

**Conclusions**

Numerous dietary supplements—including Pueraria mirifica, berberine, cannabinoids, and cesium chloride—are capable of QT prolongation via hERG blockade, CYP-mediated drug interactions, or electrolyte disturbances. These effects are particularly concerning in patients with LQTS.

**Clinical implications in patients with LQTS**

Systematically assess supplement use during clinical evaluations of QT prolongation. Evidence suggests that unnecessary supplement use should be avoided, and healthcare providers should be consulted before initiating any product. Data suggest that multiple supplements with overlapping electrophysiological effects should be avoided.

### Energy drinks

Energy drinks, marketed for their stimulant effects, contain high concentrations of caffeine, taurine, sugar, and other compounds such as guarana. Their potential to prolong the QT interval and trigger arrhythmic events has raised concerns, particularly in individuals with LQTS (TABLE 4, TABLE 5).

#### Mechanisms

Although energy drinks have been associated with QT interval prolongation, the underlying mechanisms remain incompletely understood. Caffeine itself, even at doses of up to 400 mg, does not appear to induce QTc prolongation [71], and coffee consumption has not been linked to QT prolongation [72,73]. Interestingly, Fletcher et al. [74] compared the effects of an energy drink with a control beverage with equivalent caffeine content (320 mg) and observed a significantly greater QTc prolongation (∼10 ms) in the energy drink group. This observation suggests that other constituents—such as taurine and guarana—may exert synergistic or additive effects, particularly when consumed in high volumes [75].

Further complicating safety assessment is the lack of standardized caffeine labeling. Guarana, a common additive, contains caffeine in doses of up to 60 mg/g seeds [76,77], but its caffeine content is not necessarily disclosed on product labels. Moreover, at least in the United States, many energy drinks are marketed as dietary supplements rather than conventional beverages, a classification that exempts them from regulations requiring disclosure of total caffeine content [78].

By contrast, in the European Union, caffeine content must be quantitatively disclosed when present in all high-caffeine energy drinks (>150 mg/L). This makes undeclared caffeine loads unlikely in European markets [79]. Nonetheless, the increasing complexity and variety of formulations warrant continued vigilance, especially in vulnerable populations.

#### Clinical evidence

Energy drink consumption has been linked to QT prolongation and arrhythmic events, particularly in individuals with LQTS (Table 4). Rottlaender et al. [80] reported the case of a 22-y-old female with LQT1 who developed TdP, VF, and cardiac arrest after consuming >1 L of energy drink (6 cans) within 4 h. Her QTc interval was prolonged to 526 ms during the event. Similarly, Dufendach et al. [81] described a 13-y-old girl with LQT1 (p.Gly179Ser mutation in *KCNQ1*) who developed QTc prolongation to 561 ms after consuming approximately 475 mL of energy drink daily (∼160 mg caffeine) for 2 wk.

In a retrospective study of 144 patients presenting with sudden cardiac arrest, Martinez et al. [82] identified 7 cases temporally associated with energy drink consumption. Among them were carriers of pathogenic variants in *KCNE1* (LQT5) and *CACNA1C* (LQT8), further supporting a potential role for energy drinks in unmasking or exacerbating inherited repolarization abnormalities.

Controlled trials have yielded mixed results (TABLE 4, TABLE 5). Gray et al. [83] investigated the effect of ∼500 mL of Red Bull Zero in 24 patients with LQTS. Although most participants showed no significant QTc changes, 3 individuals with a baseline QTc >480 ms and a family history of SCD exhibited QTc increases >50 ms—a threshold widely regarded as clinically significant.

**TABLE 4:** Electrocardiographic effects and arrhythmia risk associated with energy drink intake in individuals with LQTS.

Reference	Title	Article Type	Sex	Age (y)	Genetic status	Nutritional exposure	QT/arrhythmia outcome
Dufendach et al. (2012) [81]	Congenital type 1 long QT syndrome unmasked by a highly caffeinated energy drink	Case report (LoE C)	Female	13	Type 1 LQTS (*KCNQ1* G179S mutation)	≥473 mL/d (16 oz) energy drink (160 mg caffeine) for 2 wk	First ECG: QT = 420 ms, QTc = 561 ms, HR = 108 bpm 1 h later: QT = 440 ms, QTc = 557 ms, HR = 96 bpm
Rottlaender et al. (2012) [80]	Cardiac arrest due to long QT syndrome associated with excessive consumption of energy drinks	Case report (LoE C)	Female	22	*KCNQ1*	6 cans of energy drink in 4 h	TdP, VF, cardiac arrest. QTc: 526 ms (time 1) and 492 ms (time 2).
Gray et al. (2017) [83]	Cardiovascular effects of energy drinks in familial long QT syndrome: a randomized cross-over study	RCT (LoE B)	11 male, 13 female	29 ± 9	20 tested: 10 *KCNH2*, 3 *KCNQ1*, others unknown	Two cans of Red Bull Zero (∼500 mL, 160 mg caffeine, 2000 mg taurine)	No significant QTc changes overall, except in 3 individuals with severe phenotypes, who had QTc prolongation ≥50 ms
Martinez et al. (2024) [82]	Sudden cardiac arrest occurring in temporal proximity to consumption of energy drinks	Retrospective observational study (LoE C)	7 SCA survivors: 6 female	29 ± 8	Case 6: *KCNE1* mutation; Case 7: *CACNA1C* mutation; Cases 4–5: *RYR2* mutation; Case 3: *ALPK3* mutation; Cases 1–2: no mutations	Energy drink consumption 12 h to immediately before cardiac event in 5% of 144 survivors	Energy drinks might increase arrhythmia risk in predisposed individuals, although confounding factors (stress, postpartum, antibiotics) were present

Abbreviations: ALPK3, α kinase 3; CACNA1C, calcium voltage-gated channel subunit α1C; ECG, electrocardiogram; HR, heart rate; KCNE1, potassium voltage-gated channel subfamily E regulatory subunit 1; KCNH2, potassium voltage-gated channel subfamily H member 2; LoE, level of evidence; LQTS, long QT syndrome; QTc, corrected QT interval; RCT, randomized controlled trial; RYR2, ryanodine receptor 2; SCA, sudden cardiac arrest; TdP, torsade de pointes; VF, ventricular fibrillation.

**TABLE 5:** Electrocardiographic effects associated with energy drink intake in individuals without known channelopathies.

Reference	Title	Type of article	Sex	Age (y)	Genetic status	Nutritional exposure	QT/arrhythmia outcome
Buscemi et al. (2011) [72]	Acute effects of coffee on QT interval in healthy subjects	RCT (LoE B)	19 male, 21 female	21–49	Not congenital LQTS	25 mL of caffeinated espresso coffee (130 mg caffeine) vs. decaffeinated coffee (5 mg caffeine)	No statistically significant QTc changes observed
Shah et al. (2014) [88]	QTc interval prolongation with high-dose energy drink consumption in a healthy volunteer	Case report (LoE C)	Male	31	Not congenital LQTS	Two cans of Monster Energy Drink (∼950 mL, 320 mg caffeine)	QTc prolongation of 25 ms (phase A) and 22 ms (phase B)
Molnar et al. (2015) [73]	A rapid method to evaluate cardiac repolarization changes: the effect of two coffee strengths on the QT interval	Single-center trial (LoE C)	34 male, 20 female	23.4 ± 4.9	Not congenital LQTS	240 mL of Starbucks coffee (120 mg caffeine); some consumed 2 cups	Significant decrease in QTc after 30 min with 1 coffee; no QTc changes with 2 cups
Alsunni et al. (2015) [87]	Effects of energy drink consumption on corrected QT interval and heart rate variability in young obese Saudi male university students	Cross-sectional study (LoE C)	31 male	NW: 20.6 ± 0.6 OW/OB: 20.5 ± 0.7	Not congenital LQTS	5 mL/kg body weight of energy drink	Significant QTc prolongation in OW/OB group (340.2 ± 57 to 356.8 ± 54 ms); no significant QTc changes in NW group
Brothers et al. (2017) [86]	Heart rate, blood pressure and repolarization effects of an energy drink as compared to coffee	RCT (LoE B)	Protocol 1: 8 male; 7 female Protocol 2: 9 male; 6 female	27 ± 4	Not congenital LQTS	Different doses of energy drinks and coffee. Protocol 1: Energy drink and coffee volumes equivalent to 2–3 mg/kg body weight of caffeine Protocol 2: Commercially available serving sizes up to 710 mL	No significant QTc changes in either protocol
Kozik et al. (2016) [76]	Cardiovascular responses to energy drinks in a healthy population: the C-energy study	Observational study (LoE C)	12 male, 2 female	28.6 (range: 15)	Not congenital LQTS	Two cans of Monster Energy Drink (∼950 mL, 320 mg caffeine)	QTc prolongation from 423 ± 22.74 to 503 ± 24.56 ms; 8/14 subjects had QTc ≥ 500 ms; T-wave abnormalities in 9/14
Shah et al. (2016) [89]	Electrocardiographic and blood pressure effects of energy drinks and Panax ginseng in healthy volunteers: a randomized clinical trial	RCT (LoE B)	20 male, 7 female	21.6 ± 2.58	Not congenital LQTS	950 mL of energy drink vs. placebo or Panax ginseng control drink	Significant QTc increase of 6 ms with energy drink; no significant QTc changes with Panax ginseng
Fletcher et al. (2017) [74]	Randomized controlled trial of high-volume energy drink versus caffeine consumption on ECG and hemodynamic parameters	RCT (LoE B)	12 male, 6 female	26.7 ± 4	Not congenital LQTS	950 mL of energy drink (320 mg caffeine) vs. 950 mL control beverage (320 mg caffeine without energy drink ingredients)	Significant QTc prolongation of 10 ms compared with control beverage
Kozik et al. (2018) [84]	Cardiovascular responses to ENERGY drinks in a healthy population during exercise: the C-Energy-X Study	Observational study (LoE C)	10 female, 13 male	28 ± 6.9	Not congenital LQTS	Two 16-oz cans of Monster Energy Drink (∼946 mL, 320 mg caffeine)	QTc increased from 409 ± 16 to 432 ± 24 ms at rest and from 393 ± 19 to 443 ± 43 ms during exercise (*P* < 0.001); 3 subjects reached QTc ≥ 497 ms; 44% had T-wave abnormalities; 26% developed hypokalemia (lowest K^+^ = 3.2 mEq/L)
Shah et al. (2019) [85]	Impact of high-volume energy drink consumption on electrocardiographic and blood pressure parameters: a randomized trial	RCT (LoE B)	17 male, 17 female	22.1 ± 3	Not congenital LQTS	950 mL of energy drink within 60 minutes (Brand A: 304 mg caffeine; Brand B: 320 mg caffeine) vs. placebo	Brand A: QTc increase +17.9 ms (SD = 13.9); Brand B: QTc increase +19.6 ms (SD = 15.8); placebo: QTc increase +11.9 ms

Abbreviations: LoE, level of evidence; LQTS, long QT syndrome; NW, normal weight; OW/OB, overweight/obese; QTc, corrected QT interval; RCT, randomized controlled trial.

QTc prolongation has also been observed in healthy individuals consuming large volumes of energy drinks. Kozik et al. reported in 2 independent studies that the ingestion of ∼950 mL of Monster Energy Drink (320 mg caffeine) led to marked QTc increases. In the first study [76], QTc rose from 423 ± 23 ms to 503 ± 25 ms in 8 of 14 participants, with 57% exceeding the threshold of 500 ms—an incidence notably higher than in other controlled cohorts. In the second study [84], 23 healthy adults underwent rest and exercise protocols before and after energy drink ingestion. QTc increased from 409 ± 16 ms to 432 ± 24 ms at rest and from 393 ± 19 ms to 443 ± 43 ms during exercise (*P* < 0.001). Moreover, 44% of participants developed T-wave abnormalities, and 3 showed QTc values ≥497 ms.

Shah et al. [85] similarly observed QTc prolongation of 18–20 ms lasting ≤4 h after consumption of 2 different energy drink formulations (∼950 mL). This study was conducted using an industry-standard ‘thorough QT’ protocol and confirmed a statistically significant QTc increase, reinforcing concerns about the proarrhythmic potential of high-volume energy drink consumption, even in healthy volunteers [15]. By contrast, lower volumes appear less impactful. Brothers et al. [86] found no significant QTc changes in healthy volunteers consuming ≤710 mL. However, Alsunni et al. [87] noted QTc prolongation in overweight and obese individuals consuming energy drinks at 5 mL/kg, with no effect in normal-weight participants.

Importantly, over 30 deaths attributed to energy drinks have been reported to the FDA, and cases of TdP have occurred in individuals with LQTS after energy drink use [15]. Despite these risks, energy drinks are often marketed without medical oversight, and their consumption remains widespread among adolescents, athletes, and young adults. As highlighted by Woosley [15], this widespread, unsupervised use of bioactive, poorly regulated products poses a significant and emerging risk to individuals with congenital or acquired susceptibility to repolarization abnormalities.

**Conclusions**

Energy drinks have been implicated in QT prolongation and arrhythmic events, especially among individuals with LQTS. Although the mechanisms remain partially elucidated, the combination of caffeine with other bioactive compounds likely contributes to proarrhythmic risk. Even in healthy individuals, high-volume intake may lead to transient QTc prolongation, justifying clinical caution.

**Clinical implications in patients with LQTS**

It would be useful to evaluate energy drink consumption at all visits. Evidence suggests that energy drinks should be avoided.

## Nutritional Interventions for Patients with LQTS

Although dietary factors may exacerbate QT prolongation, nutritional interventions offer a potential strategy to mitigate arrhythmic risk in LQTS. Emerging evidence indicates that targeted nutritional approaches—such as potassium supplementation—can directly modulate ion channel function and indirectly optimize electrolyte and metabolic homeostasis. By enhancing hERG channel stability and improving repolarization, these interventions may reduce QT prolongation and lower arrhythmic vulnerability. This section reviews the mechanistic rationale, clinical efficacy, and limitations of such nutritional strategies tailored to specific LQTS genotypes.

### Potassium supplementation

The rationale for using potassium supplementation as adjunctive therapy in LQTS originated from early biophysical studies demonstrating that elevated extracellular potassium enhances hERG channel function [90]. Seminal work by Sanguinetti et al. [91] established that I_Kr_ current is directly proportional to extracellular potassium concentration [91], such that reductions in extracellular potassium acutely decrease I_Kr_ amplitude in cardiomyocytes [92] and hERG current in heterologous expression systems [90]. Studies in rabbit hearts further showed that the cell surface density of hERG channels is dynamically regulated by extracellular potassium within a physiologically relevant range, with rapid internalization and degradation of hERG channels after potassium depletion [93]. More recent evidence suggests that potassium binding at the mouth of the external pore of the hERG channel is essential to maintain its functional conformation and membrane stability [94]. A direct relationship between ion permeability and membrane stability was demonstrated for external permeant cations (K^+^, Rb^+^, and Cs^+^), supporting the hypothesis that these ions interact with site(s) within the permeation pathway to stabilize the channel. This interpretation was further reinforced by the observation that specific point mutations in the selectivity filter and pore helix (e.g., the p. Ser624Thr in *KCNH2*) abrogates the requirement for extracellular potassium to preserve hERG function and membrane localization [94].

From a mechanistic standpoint, these findings support the concept that exogenous potassium administration may partially restore impaired I_Kr_ function in LQT2.

#### Evidence for clinical efficacy and limitations

Early clinical studies explored the potential of high-dose potassium supplementation in patients with LQT2, usually in conjunction with spironolactone (Table 6). Compton et al. [95] administered intravenous potassium chloride (20 mEq/h) combined with oral potassium (60 mEq every 2 h) and high-dose spironolactone (200 mg initially, then 100 mg every 2 h) to patients with LQT2, delivering a total potassium dose of 162 ± 67 mEq. This aggressive protocol achieved a 24% QTc reduction (from 627 ± 90 ms to 469 ± 23 ms), with serum potassium increasing on average by ≥1.4 ± 0.7 mEq/L to ∼5.5 mEq/L.

**TABLE 6:** Effects of potassium supplementation on QT interval in patients with long QT syndrome.

Reference	Article type	Sex	Age (y)	Genetic status	Nutritional exposure	QT/arrhythmia outcome
Compton et al. (1996) [95]	Clinical trial (LoE C)	4 male, 3 female	19–48 (34 ± 12)	LQT2	IV potassium chloride: 20 mEq/h Oral potassium: 60 mEq every 2 h Total potassium: 162 ± 67 mEq Spironolactone: 200 mg initial dose, then 100 mg every 2 h	QTc reduction by 24% (−159 ± 94 ms) from 627 ± 90 at baseline. QT dispersion: 133 ± 62 ms → 42 ± 28 ms T-wave normalization: 6/7 patients
Etheridge et al. (2003) [96]	Clinical trial (LoE C)	8 patients; sex not specified	11–52 (35 ± 17)	LQT2	Oral potassium: 3.3 ± 1.5 mEq/kg/d KCl Spironolactone: 3.5 ± 1.2 mg/kg/d	QTc reduction: 526 ± 94 ms → 423 ± 36 ms T-wave normalization: 4/8 patients Hyperkalemia (>6.0 mEq/L): 3/8 patients
Marstrand et al. TriQarr Study (2021) [98]	Clinical trial (LoE C)	9 female, 1 male (high-dose+ group)	41.5 ± 6.9	5 LQT1/5 LQT2	100 mg spironolactone + 40 mEq KCl/d	Plasma K^+^: 4.08 → 4.48 mEq/L QTcF (rest): 472 ± 8 → 469 ± 8 ms Side effects: 4/10 (mild)

Abbreviations: IV, intravenous; LoE, level of evidence.

Similarly, Etheridge et al. [96] demonstrated QTc shortening of 103 ms using chronic high-dose weight-adjusted oral potassium (3.3 ± 1.5 mEq/kg/d potassium chloride) and spironolactone (3.5 ± 1.2 mg/kg/d), corresponding to approximately 231 mEq potassium/d and 245 mg spironolactone/d for a 70-kg individual. This regimen increased serum potassium by 1.2 mEq/L. However, 3 of 8 patients developed hyperkalemia (>6.0 mEq/L), underscoring safety concerns [96]. An unpublished trial by Mason et al. in November 2002 adopted a dosing regimen like the one used by Compton et al. but was prematurely terminated due to concerns about hyperkalemia [97].

A more recent study by Marstrand et al. [98] assessed the feasibility of moderate-dose potassium supplementation in a mixed cohort of patients with LQT1 and LQT2. In this short-term intervention, daily administration of 40 mEq of potassium chloride with 100 mg of spironolactone for 1 wk increased serum potassium by only 0.4 mEq/L—substantially less than what was achieved in earlier high-dose regimens. Importantly, this modest increase did not result in QTc shortening [98,107,109].

Overall, available evidence (Table 6) suggests that a meaningful QTc shortening effect requires a serum potassium increase of ≥1.0 mmol/L, a threshold difficult to achieve without resorting to high-dose supplementation protocols [96]. These regimens, however, present important limitations. The pill burden alone can be substantial, sometimes exceeding 20 tablets daily, as reported by Marstrand et al. [98], limiting long-term patient adherence. Moreover, the combination of high-dose potassium with spironolactone, frequently administered together with βB, raises concerns regarding hypotension, renal dysfunction, and hyperkalemia.

In addition, although preclinical studies suggest a sigmoidal relationship between serum potassium and arrhythmic risk, with a sharp increase in risk below 3.5 mmol/L, there is no clinical evidence supporting additional benefit from raising potassium levels above the normal physiologic range (∼4.0–5.0 mmol/L) [98,99]. This raises questions about the justification for aggressive supplementation, especially considering the associated safety risks.

Finally, another important consideration is the heterogeneity of response among different LQTS genotypes. Although LQT2 is mechanistically predisposed to benefit from potassium supplementation because of direct effects on hERG channel function, available data, including the Marstrand et al. study [98], suggest that QTc shortening is not consistently achieved in clinical settings, even within LQT2 cohorts. Moreover, the inclusion of mixed-genotype populations in most trials, especially those combining LQT1 and LQT2, may obscure potential genotype-specific effects. For LQT1, clinical data remain limited and do not currently support the use of potassium supplementation.

Given these limitations, potassium supplementation should be considered only in selected patients with LQT2, particularly those with recurrent arrhythmias despite optimal medical therapy. When used, it should be accompanied by close monitoring of serum potassium levels and renal function to mitigate risk of adverse events. Although potentially beneficial in carefully selected cases, chronic high-dose potassium supplementation cannot be recommended as a routine strategy.

**Conclusions**

Despite its strong mechanistic rationale, potassium supplementation has demonstrated limited clinical efficacy in reducing QTc in LQT2, especially when administered at moderate doses. High-dose regimens requiring ≤20 pills per day, although more effective in achieving target potassium levels, are constrained by safety concerns and poor tolerability. Therefore, potassium supplementation is not currently recommended for widespread clinical use but may be considered in specific patients with LQT2 under careful supervision.

**Clinical implications in patients with LQTS**

Evidence suggests that potassium supplementation may be considered in selected patients with LQT2 with recurrent arrhythmias despite optimized therapy, especially when serum potassium is consistently <4.0 mEq/L. However, no data exist to support the chronic use of high-dose protocols because of poor tolerability and risk of adverse events.

### Dietary recommendations for Andersen-Tawil syndrome (LQT7)

Andersen-Tawil syndrome (ATS; also referred to as LQT7) is a rare autosomal dominant channelopathy caused in ∼60% of cases by loss-of-function or dominant-negative mutations in *KCNJ2*, encoding the K_ir_2.1 inward-rectifier potassium channel, which plays a critical role in stabilizing the resting membrane potential of skeletal and cardiac muscle cells [4]. The syndrome presents with a variable combination of periodic paralysis (PP), ventricular arrhythmias, and dysmorphic features, with the full triad observed in 40%–60% of genetically confirmed patients [100,101]. The cardiac phenotype results from reduced I_K1_ current, which delays terminal repolarization and manifests electrocardiographically with prominent U waves. Although initially classified among the LQTSs [102], QT prolongation is not mandatory for diagnosis [103].

The arrhythmogenic substrate involves the presence of delayed afterdepolarizations, emerging during hypokalemia because of abnormal calcium extrusion via the Na^+^/Ca^2+^ exchanger [102]. In skeletal muscle, I_Kir_ deficiency leads to K-dependent depolarization of the resting membrane potential, which promotes sodium channel inactivation and episodic paralysis [104]. Interestingly, ATS1 is characterized by a unique bidirectional potassium sensitivity: both hypokalemia and hyperkalemia may precipitate weakness. Hypokalemia paradoxically worsens depolarization because of insufficient outward K current to balance the normal inward Cl current, whereas the mild depolarization normally produced by hyperkalemia is amplified because the attenuated outward I_Kir_ is unable to balance subthreshold persistent inward sodium current (i.e., window current), promoting additional depolarization [104].

**Conclusions**

Dietary management plays a supportive role in reducing symptom burden, particularly in patients with PP. Episodes may be triggered by specific macronutrient compositions: most commonly carbohydrate-rich or potassium-modulating foods, depending on the individual’s susceptibility to hypoPP or hyperPP.

**Clinical implications in patients with ATS1**

Evidence suggests that dietary strategies should be individualized based on the patient’s predominant potassium disturbance (i.e., hypokalemic compared with hyperkalemic episodes).

For hypokalemic episodes, evidence favors a low-sodium, low-carbohydrate diet, along with potassium supplementation. On the contrary, data suggest that eating frequent meals with sufficient carbohydrates can help prevent attacks during hyperkalemic episodes.

#### Muscular manifestations and potassium dysregulation

PP in ATS presents as recurrent episodes of muscle weakness or paralysis, with highly variable frequency and duration among affected individuals [100,105]. These episodes are associated with fluctuations in potassium levels, manifesting as hypokalemic (hypoPP), hyperkalemic (hyperPP), or normokalemic PP. The specific potassium imbalance in individual patients dictates both symptomatology and therapeutic approaches, and tailored nutritional strategies aim to mitigate triggers, prevent attacks, and manage acute episodes [100,105]. Importantly, because patients with ATS may experience either hypoPP or hyperPP episodes, dietary modifications should be personalized based on their specific potassium disturbances [100].

#### Hypokalemic periodic paralysis

Well-established dietary triggers of hypoPP [100,105] include high-carbohydrate meals, irregular meal patterns, large meals, alcohol consumption, and eating late in the evening. The specific carbohydrate composition that provokes paralysis remains unclear, but reported triggers include chocolate, sweets, sugary carbonated beverages, snacks, pasta, and foods high in both carbohydrates and sodium, such as fast food or Chinese cuisine. Simple carbohydrates, particularly those in sugary beverages and sweets, may have a stronger impact on triggering episodes.

Management strategies should distinguish between prophylactic dietary measures and acute intervention:

• *Preventive dietary modifications*: A low-sodium, low-carbohydrate diet is generally recommended. Potassium chloride-based salt substitutes may benefit patients who experience symptoms linked to sodium intake [106]. In addition, hyperosmolar states, including hyperglycemia and dehydration, should be avoided. Carbohydrate-heavy meals should preferably be avoided, particularly in the late evening.
• *Acute dietary management*: Potassium supplementation is commonly utilized, although slow-release potassium formulations should be avoided during acute attacks to ensure rapid correction of potassium levels.

#### Hyperkalemic periodic paralysis

Nutritional triggers for hyperPP [100,105] include fasting, skipping meals, potassium-rich foods or supplements, alcohol consumption, cold foods or drinks, and certain unspecified dietary factors.
Key dietary recommendations include:
• *Preventive measures*: Frequent meals throughout the day, ensuring a balanced intake of carbohydrates, can help stabilize potassium levels. Identifying and avoiding individual dietary triggers is crucial.
• *Acute management*: Carbohydrate consumption is recommended to counteract episodes of hyperkalemia, with no significant differences observed between solid and liquid carbohydrate sources. It is also reported that abundant hydration helps in resolving symptoms.

#### Individual variability in dietary triggers

Although these dietary triggers have been described in the literature, individual variability in ATS remains significant. Personalizing dietary recommendations based on patient-reported triggers is an essential component of management. In a recent cohort study of 35 patients with ATS, carbohydrate ingestion was identified as a trigger in 6 cases, whereas fasting was implicated in 1 case. Among the 28 patients experiencing PP, ∼20% exhibited symptoms associated with carbohydrate intake [101].

### Dietary recommendations for Timothy syndrome (LQT8)

Timothy syndrome (TS) is among the most severe forms of LQTS, caused by gain-of-function mutations in the *CACNA1C* gene, which encodes the L-type calcium channel Ca_V_1.2. The most studied mutation, G406R in the alternatively spliced exon 8A, leads to impaired voltage-dependent inactivation of the channel, resulting in prolonged cardiac repolarization and severe QT prolongation. TS is a multisystem disorder characterized by syndactyly, autism spectrum disorders, and intermittent hypoglycemia, which exacerbates arrhythmias and contributes to SCD [6,107].

Currently, 2 forms of TS have been identified: TS1, characterized by a G406R mutation in exon 8A, and TS2, characterized by a G406R mutation in exon 8 [108]. Other *CACNA1C* variants have been identified, and LQT8 can occur independently, without the multisystemic features characteristic of TS [108].

#### Hypoglycemia in TS

Hypoglycemia in TS has been widely documented (Table 7), with initial reports by Splawski et al. [6] noting its presence in 36% of patients with TS, including 2 deaths attributed to hypoglycemia. Subsequent studies have further explored its clinical relevance and mechanisms.

**TABLE 7:** Reported cases of hypoglycemia in Timothy syndrome and associated QT outcomes.

Reference	Type of article	Sex	Age	Genetic status	Type of LQTS	Data on hypoglycemia
Splawski et al. (2004) [6]	Case report (LoE C)	9 male, 8 female	—	G406R mutation on exon 8A of *CACNA1C* gene	LQT8	Hypoglycemia in 36% of patients; 2 deaths caused by arrhythmias associated with hypoglycemia
Dufendach et al. (2018) [114]	Multicenter study (LoE C)	88% male	Median: 6.4 y	—	LQT8	Hypoglycemia in 7/17 patients; 11 cases of SCD or ACA; 2 SCD/ACA linked to hypoglycemia; 4 VF deaths (2/4 with hypoglycemia).
Kummer et al. (2022) [110]	Case report + retrospective analysis (LoE C)	Female	17 y	CACNA1C c.1679T>C, p.L566P (CaV1.2 L566P)	*CACNA1C* mutation without QT prolongation	First case of congenital hyperinsulinism with a *CACNA1C* mutation; retrospective review of 5 patients’ medical records identified variable hypoglycemia presentations (hyperinsulinemic or ketosis-associated).
Matthews et al. (2023) [109]	Observational cohort study (LoE C)	26 male, 18 female	8 mo–3 y	G406R mutation on exon 8A of *CACNA1C* gene	LQT8	Neonatal hypoglycemia in 7/44 patients; 16 deaths (primary causes: SCD, sepsis, hypoglycemia); 2 deaths in patients with ICD caused by hypoglycemia.

Abbreviations: ACA, aborted cardiac arrest; CACNA1C, calcium voltage-gated channel subunit α1C; ICD, implantable cardioverter defibrillator; LoE, level of evidence, LQTS, long QT syndrome; SCD, sudden cardiac death; VF, ventricular fibrillation.

A multicenter retrospective cohort study by Dufendach et al. [81], collecting data from 12 international pediatric centers on patients with TS diagnosed between 1994 and 2016, identified hypoglycemia as a frequent and potentially life-threatening complication. In this cohort of 17 patients, 7 experienced documented hypoglycemic episodes, and 72% of all SCD or aborted cardiac arrest (ACA) events occurred in the context of either hypoglycemia or general anesthesia. Importantly, 11 of 17 patients experienced either ACA or died from SCD, with arrhythmias clearly associated with hypoglycemia documented in ≥2 cases, including 1 fatality despite the presence of an ICD. VF was consistently identified as the terminal rhythm when available. Notably, among the 4 VF-related deaths, 2 were directly associated with hypoglycemic episodes [120]. Similarly, an international neonatal cohort study by Matthews et al. [5] identified hypoglycemia in 7 of 44 (16%) cases. Hypoglycemia contributed to 2 deaths in patients with ICDs and was a major cause of early morbidity alongside SCD and sepsis [109].

#### Mechanistic insights

The initial hypothesis that hyperinsulinism underlies hypoglycemia in TS was proposed by Splawski et al. [6] based on the observation that “in the pancreas, Ca^2+^ mediates insulin secretion by pancreatic β-cells. Episodic dysfunction of Ca_V_1.2 signaling likely accounts for the intermittent hypoglycemia.” However, this hypothesis has not been investigated further for many years.

In 2022, a retrospective review examined 5 patients with the *CACNA1C* G406R mutation located in exon 8A or 8 who exhibited hypoglycemia [110]. The analysis revealed that some of these patients had hyperinsulinemic hypoglycemia, whereas others displayed ketosis-associated hypoglycemia. Because the presence of ketone bodies excludes hyperinsulinism as the underlying mechanism, this finding suggested that alternative mechanisms contribute to hypoglycemia in TS.

This hypothesis was further explored by Matsui et al. [111] in a TS2 murine model, which provided direct evidence for insulin-independent pathways. In these mice, hypoglycemia was unexpectedly accompanied by hypoinsulinemia. It was linked instead to multiple factors, including impaired counterregulatory hormone responses (e.g., reduced glucagon secretion), glycosuria, and abnormal hypothalamic regulation of glucose metabolism. Additionally, the authors described a TS1 patient with fasting hypoglycemia and ketosis, further supporting the notion that hyperinsulinism is not the sole driver of hypoglycemia in TS. These findings highlight a complex interplay between central nervous system dysfunction, hormone dysregulation, and peripheral glucose metabolism in the pathogenesis of TS-related hypoglycemia [111].

**Conclusions**

Hypoglycemia is a significant risk factor for arrhythmias and SCD in individuals with Timothy Syndrome. The mechanisms underlying hypoglycemia in TS are diverse, involving both insulin-dependent and insulin-independent pathways. These complexities necessitate tailored dietary and pharmacological interventions.

**Clinical implications in patients with TS**

Given the scarcity of specific dietary guidelines for managing hypoglycemia in patients with TS within the scientific literature, insights from the Timothy Syndrome Foundation’s website offer practical suggestions [112]:

• **Frequent, small meals:** Encourage consumption of small, balanced meals throughout the day to maintain stable glucose levels and prevent prolonged fasting, a common trigger for hypoglycemia.
• **Postexercise carbohydrate intake:** Advise carbohydrate ingestion after physical activity to replenish energy stores and prevent hypoglycemic episodes**.**
• **Bedtime snacks:** Suggest snacks rich in slow-digesting carbohydrates before bedtime to prevent overnight hypoglycemia**.**
• **Balanced macronutrient intake:** Ensure meals contain appropriate amounts of carbohydrates, proteins, and fats to provide sustained energy release and minimize rapid glucose fluctuations**.**
• **Management of hyperinsulinism:** In TS cases with confirmed hyperinsulinism, following existing nutritional guidelines for hypoglycemia management is advisable [113].

#### Hypoglycemia in other forms of LQTS

In addition to TS, episodes of hypoglycemia have been observed in patients with LQT1 and LQT2 [115,116]. Torekov et al. [115] and Hyltén-Cavallius et al. [116] investigated glucose homeostasis in these subtypes using oral glucose tolerance tests (OGTTs), continuous 24-h glucose monitoring, and symptom questionnaires. Both studies documented lower blood glucose levels, with some patients experiencing true hypoglycemia, defined as plasma glucose <50 mg/dL and associated with clinical symptoms [115,116].

##### Mechanisms of hypoglycemia in LQT1 and LQT2

Unlike TS, in which hypoinsulinemia may be present [111], patients with LQT1 and LQT2 exhibit increased insulin secretion. In LQT1, Rosengren et al. [117] and Yamagata et al. [118] demonstrated that inhibition of KCNQ1 enhances insulin exocytosis. Torekov et al. [115] extended these findings by showing that other glucose-regulating hormones played a minor role in the hypoglycemia observed in patients with LQT1. In LQT2, Hyltén-Cavallius et al. [116] observed that pharmacologic blockade of hERG channels in animal models resulted in increased insulin secretion and reduced glycemic levels. Furthermore, knockdown of hERG in intestinal L cells led to amplified insulin and GLP-1 secretion in response to glucose [116]. They also observed that patients with LQT2 exhibited reduced glucagon secretion (similar to TS [111]) and increased secretion of GLP-1 and GIP, which could lead to excessive insulin release and glucose fluctuations.

##### QT prolongation and glucose instability

Both studies reported QT prolongation after OGTTs. In LQT1, an additional QTc prolongation of 10 ± 2 ms was noted [115], whereas in LQT2, the maximum QTc increase reached 25.5 ± 3.6 ms [116].

Interestingly, patients with LQT2 exhibited QT prolongation during both hyperglycemia and hypoglycemia, indicating that glucose fluctuations may exacerbate arrhythmic risk.

##### Nutritional implications

Patients with LQT1 and LQT2 experienced postprandial reactive hypoglycemia, particularly 3–5 h after meals in LQT1 [115], reinforcing the need for dietary strategies aimed at maintaining glycemic stability.

Although the mechanisms differ from those in postbariatric hypoglycemia, hyperinsulinemia is a shared feature [119,120]. In postbariatric patients, reducing carbohydrate intake to <30 g per meal and <15 g per snack has been effective in mitigating hypoglycemic episodes [121]. However, in LQTS-related hypoglycemia, a more balanced approach is recommended, ensuring carbohydrate intake accounts for ≥45% of total daily energy [122] while minimizing rapid glycemic excursions.

**Conclusions**

In LQT1 and LQT2, genotype-specific alterations in insulin and counter-regulatory hormone secretion contribute to postprandial hypoglycemia. In LQT2, both hypo- and hyperglycemia have been associated with QTc prolongation, suggesting that glycemic fluctuations—not just absolute values—can increase arrhythmic risk. Nutritional strategies should therefore prioritize glycemic stability through balanced, individualized dietary planning.

**Clinical implications in patients with LQTS**

Postprandial hypoglycemia should be recognized as a potential arrhythmogenic trigger.

Dietary interventions should aim to:

• Prevent glycemic excursions through frequent, evenly spaced meals.
• Emphasize complex carbohydrates and avoid high-glycemic index foods.
• Include protein and unsaturated fats to blunt postprandial insulin peaks.
• Distribute carbohydrate intake evenly, aiming for ≥45% of total energy from carbohydrates to maintain glycemic and electrophysiologic stability.

## Conclusions

Diet and metabolism emerge as relevant but underappreciated contributors to arrhythmic risk modulation in LQTS. Beyond the canonical view of arrhythmias as purely genetically determined, the available evidence highlights how nutritional exposures interact dynamically with an already vulnerable repolarization reserve, sometimes tipping the balance toward life-threatening arrhythmias.

This interaction is mechanistically diverse, involving ion channel modulation, altered autonomic responses, electrolyte imbalances, and changes in drug metabolism. The significance of these factors becomes particularly relevant in inherited channelopathies where genetic defects have already constrained repolarization adaptability. Importantly, the nutritional field presents both risk and opportunity. On one hand, inappropriate or unrecognized exposures (e.g., licorice, grapefruit, energy drinks, and herbal supplements) may contribute to the clinical expression of the disease or even precipitate arrhythmic events. On the contrary, tailored dietary interventions could represent a feasible and safe adjunctive strategy to reduce arrhythmic burden.

However, translating mechanistic plausibility into clinically effective interventions remains challenging. Studies on potassium supplementation, for example, illustrate the gap between strong pathophysiologic rationale and the limited benefit achievable under safe clinical conditions. Moving forward, several unmet needs should be addressed. First, the arrhythmogenic potential of dietary compounds needs to be quantified in a systematic and genotype-specific manner. From a practical point of view, it would be useful to inquire about food supplements during medical visits and to educate patients about the potential benefits and harm that may be associated with various nutritional factors, including but not limited to supplements. Second, the design of controlled clinical trials assessing the effectiveness and safety of nutritional interventions in reducing arrhythmic events is essential.

Finally, nutritional factors should be integrated into risk stratification models, particularly for patients with borderline phenotypes or unexplained arrhythmias. Until such data are available, systematic assessment of dietary habits and metabolic status, combined with cautious, individualized recommendations, should become the standard of care for patients with LQTS.

## Methods

This narrative review was designed to analyze and summarize current knowledge on the relationship between nutrition and arrhythmic risk in patients with LQTS. A comprehensive literature search was conducted in PubMed up to December 2024. The search strategy combined concepts related to LQTS and arrhythmic outcomes with a wide range of dietary and metabolic exposures, encompassing specific foods, beverages, supplements, electrolytes, and metabolic conditions of interest. Additional articles were identified by manual screening of the bibliographies of relevant studies and clinical guidelines, as well as through targeted follow-up searches to capture emerging or related evidence. The review focused on human studies, including case reports, case series, observational studies, and clinical trials. Experimental and in vitro studies were included selectively when they provided mechanistic insights that could support or contextualize clinical findings. Studies were excluded if they lacked relevance to both arrhythmic outcomes and nutritional factors. To aid interpretation, an evidence hierarchy was applied: randomized controlled trials were considered the highest level of evidence, followed by observational studies, and finally, individual case reports or case series. This evidence hierarchy was classified using levels of evidence as in the European Society of Cardiology guidelines. Data from all relevant clinical studies were systematically summarized in structured tables to facilitate critical appraisal and practical consultation by clinicians and nutrition professionals.

## Limitations

This work represents the first comprehensive attempt to summarize the influence of dietary and nutritional factors on arrhythmic risk in individuals with LQTS but is subject to the inherent limitations of a narrative review. The rarity of the disease and the scarcity of large, prospective trials limited the availability of high-level evidence, with much of the literature analyzed consisting of case reports, case series, or small observational studies. The narrative format allowed inclusion of heterogeneous evidence and integration of mechanistic data, but no formal bias assessment or meta-analysis was performed. Although the literature search was extensive, including manual bibliography screening and targeted follow-up searches, it is possible that some relevant studies were not identified, also because the review focused on studies published in the English language. Nevertheless, the assembled body of evidence provides a clinically meaningful synthesis to inform practice and guide future research.

## Author contributions

The authors’ responsibilities were as follows – AM: designed the review; AM, MF: conducted literature analysis and drafted the manuscript; AM, DK, AT: revised the manuscript; DK, AT, SGP: provided critical revisions; AM: had primary responsibility for the final content; and all authors: read and approved the final manuscript.

## Declaration of AI and AI-assisted technologies in the writing process

During the preparation of this manuscript, the authors occasionally used OpenAI’s large language model ChatGPT to assist with proofreading and formatting consistency. No large language model has been used for content generation or data analysis. All content was critically reviewed and edited by AM and MF, who take full responsibility for its accuracy and interpretation.

## Funding

This work was supported by the *Ricerca Corrente* *funding scheme* of the Italian Ministry of Health. The funding source had no role in the study design, data analysis, writing, or decision to publish this manuscript.

## Conflict of interest

The authors report no conflicts of interest.
